# Supplementary material for: Evaluating Self-Directed Behaviours and Their Association with Emotional Arousal across Two Cognitive Tasks in Bonobos (Pan paniscus)
Source: Animals (Basel). 2022 Nov 1;12(21):3002. doi: 10.3390/ani12213002 (PMC9656221; doi:10.3390/ani12213002)
Supplement: Supplementary file 1 [file animals-12-03002-s001.zip › Supplementary Tables.pdf]

Evaluating self-directed behaviours and their association with emotional arousal across two cognitive tasks in bonobos (*Pan paniscus*)

Daan W. Laméris, Jonas Verspeek, Marina Salas, Nicky Staes, Jonas R. R. Torfs, Marcel Eens, Jeroen M. G. Stevens

Supplementary materials

Table S1: Model outputs testing the effects of condition and stimulus type on the rate of SDBs in Study 1

Nose wipe ~ condition + stimulus

	Chisq	Df	*P*
Condition	0.554	2	0.758
Stimulus	3.393	2	0.183

Fixed factor	Contrast	Estimate	SE	df	t-ratio	*P*
Stimulus	Averted – Control	0.001	0.002	5864	0.379	1.000
	Averted – Direct	-0.002	0.001	5864	-1.418	0.469
	Control - Direct	-0.003	0.002	5864	-1.698	0.269
Condition	Pre-baseline – Post-baseline	0.0004	0.001	5744	0.236	1.000
	Pre-baseline – Fusion	-0.0008	0.001	5719	-0.554	1.000
	Post-baseline - Fusion	-0.001	0.002	5870	-0.719	1.000

Gentle self-scratch ~ condition + stimulus

	Chisq	Df	*P*
Condition	0.746	2	0.689
Stimulus	6.759	2	0**.034**

Fixed factor	Contrast	Estimate	SE	df	t-ratio	*P*
Stimulus	Averted – Control	-0.002	0.0008	5864	-2.392	**0.050**
	Averted – Direct	-0.002	0.0008	5864	-2.016	0.132
	Control - Direct	0.0004	0.0008	5864	0.524	1.000
Condition	Pre-baseline – Post-baseline	-0.0005	0.0008	5710	-0.582	1.000
	Pre-baseline – Fusion	0.0003	0.0008	5679	0.354	1.000
	Post-baseline - Fusion	0.0008	0.0009	5870	0.853	1.000

Rough self-scratch ~ condition + stimulus

	Chisq	Df	*P*
Condition	1.349	2	0.510
Stimulus	1.737	2	0.420

Fixed factor	Contrast	Estimate	SE	df	t-ratio	*P*
Stimulus	Averted – Control	0.0007	0.0006	5865	1.179	0.715
	Averted – Direct	0.0006	0.0006	5865	1.070	0.854
	Control - Direct	-0.0001	0.0006	5865	-0.188	1.000
Condition	Pre-baseline – Post-baseline	6.61e-04	0.0006	4384	1.112	0.799
	Pre-baseline – Fusion	8.34e-05	0.0006	4203	0.141	1.000
	Post-baseline - Fusion	-5.77e-04	0.0007	5860	-0.883	1.000

Table S2: Model outputs testing the effects of stimulus type on the rate of SDBs in Study 2 – Experiment 1

Nose wipe ~ stimulus				Chisq	Df	*P*
			Stimulus	1.027	2	0.599
Fixed factor	Contrast	Estimate	SE	df	t-ratio	*P*
Stimulus	Congruent – Control	-0.003	0.004	1217	-0.899	1.000
	Congruent – Incongruent	-0.004	0.004	1217	-0.882	1.000
	Control – Incongruent	-0.0002	0.004	1217	-0.064	1.000
Gentle self-scratch ~ stimulus				Chisq	Df	*P*
			Stimulus	1.565	2	0.457
Fixed factor	Contrast	Estimate	SE	df	t-ratio	*P*
Stimulus	Congruent – Control	-0.001	0.001	1218	-1.070	0.855
	Congruent – Incongruent	-0.002	0.001	1218	-1.127	0.780
	Control – Incongruent	-0.0002	0.001	1218	-0.160	1.000
Rough self-scratch ~ stimulus				Chisq	Df	*P*
			Stimulus	3.083	2	0.214
Fixed factor	Contrast	Estimate	SE	df	t-ratio	*P*
Stimulus	Congruent – Control	-0.002	0.001	1218	-1.125	0.782
	Congruent – Incongruent	0.0008	0.001	1218	0.515	1.000
	Control – Incongruent	0.002	0.001	1218	1.687	0.276

Table S3: Model outputs testing the effects of stimulus type on the rate of SDBs in Study 2 – Experiment 2

Nose wipe ~ stimulus				Chisq	Df	*P*
			Stimulus	8.447	3	**0.034**
Fixed factor	Contrast	Estimate	SE	df	t-ratio	*P*
Stimulus	Blank – Negative	0.004	0.004	1817	1.035	1.000
	Blank – Neutral	0.009	0.004	1817	2.108	0.211
	Blank – Positive	0.012	0.004	1817	2.655	**0.048**
	Negative – Neutral	0.005	0.005	1817	0.995	1.000
	Negative – Positive	0.007	0.005	1817	1.504	0.796
	Neutral - Positive	0.002	0.005	1817	0.508	1.000
Gentle self-scratch ~ stimulus				Chisq	Df	*P*
			Stimulus	2.006	3	0.571
Fixed factor	Contrast	Estimate	SE	df	t-ratio	*P*
Stimulus	Blank – Negative	0.002	0.002	1817	0.957	1.000
	Blank – Neutral	0.002	0.002	1817	0.903	1.000
	Blank – Positive	0.002	0.002	1817	1.315	1.000
	Negative – Neutral	-0.00009	0.002	1817	-0.050	1.000
	Negative – Positive	0.0006	0.002	1817	0.333	1.000
	Neutral - Positive	0.0007	0.002	1817	0.382	1.000
Rough self-scratch ~ stimulus				Chisq	Df	*P*
			Stimulus	2.283	3	0.516
Fixed factor	Contrast	Estimate	SE	df	t-ratio	*P*
Stimulus	Blank – Negative	-0.002	0.001	1818	-1.419	0.937
	Blank – Neutral	-0.0003	0.001	1818	-0.262	1.000
	Blank – Positive	-0.0002	0.001	1818	-0.172	1.000
	Negative – Neutral	0.001	0.001	1817	1.071	1.000
	Negative – Positive	0.002	0.001	1817	1.153	1.000
	Neutral - Positive	0.0001	0.001	1817	0.083	1.000

Table S4: Model outputs testing the effects of stimulus type on the rate of SDBs in Study 2 – Experiment 3

Nose wipe ~ stimulus				Chisq	Df	*P*
			Stimulus	5.439	3	0.142
Fixed factor	Contrast	Estimate	SE	df	t-ratio	*P*
Stimulus	Blank – Negative	0.001	0.005	1670	0.212	1.000
	Blank – Neutral	-0.010	0.005	1670	-1.996	0.276
	Blank – Positive	-0.001	0.005	1670	-0.249	1.000
	Negative – Neutral	-0.011	0.005	1670	-2.045	0.246
	Negative – Positive	-0.002	0.005	1670	-0.427	1.000
	Neutral - Positive	0.008	0.005	1670	1.620	0.633
Gentle self-scratch ~ stimulus				Chisq	Df	*P*
			Stimulus	3.872	3	0.276
Fixed factor	Contrast	Estimate	SE	df	t-ratio	*P*
Stimulus	Blank – Negative	-0.001	0.002	1670	-0.715	1.000
	Blank – Neutral	-0.0002	0.002	1670	-0.087	1.000
	Blank – Positive	0.003	0.002	1670	1.341	1.000
	Negative – Neutral	0.001	0.002	1670	0.587	1.000
	Negative – Positive	0.004	0.002	1670	1.905	0.342
	Neutral - Positive	0.003	0.002	1670	1.330	1.000
Rough self-scratch ~ stimulus				Chisq	Df	*P*
			Stimulus	3.297	3	0.348
Fixed factor	Contrast	Estimate	SE	df	t-ratio	*P*
Stimulus	Blank – Negative	0.0002	0.001	1670	0.197	1.000
	Blank – Neutral	0.001	0.001	1670	1.212	1.000
	Blank – Positive	0.002	0.001	1670	1.553	0.724
	Negative – Neutral	0.001	0.001	1670	0.938	1.000
	Negative – Positive	0.002	0.001	1670	1.255	1.000
	Neutral - Positive	0.0004	0.001	1670	0.324	1.000

Daan W. Laméris, Jonas Verspeek, Marina Salas, Nicky Staes, Jonas R. R. Torfs, Marcel Eens, Jeroen M. G. Stevens

Table S5: Rates of SDBs per trial per second for the subjects across the two studies

	Busira		Habari	
	Study 1	Study 2	Study 1	Study 2
Nose wipe	0.018 ± 0.002	0.036 ± 0.002	0.009 ± 0.001	0.011 ± 0.001
Rough self-scratch	0.0015 ± 0.0006	0.0026 ± 0.0007	0.0016 ± 0.0006	0.0004 ± 0.0002
Gentle self-scratch	0.0051 ± 0.0011	0.0065 ± 0.0012	0.0043 ± 0.001	0.0028 ± 0.00061
	Kianga		Kikongo	
	Study 1	Study 2	Study 1	Study 2
Nose wipe	0.017 ± 0.002	NA	0.007 ± 0.002	NA
Rough self-scratch	0.0018 ± 0.0005	NA	0.0001 ± 0.0001	NA
Gentle self-scratch	0.0075 ± 0.0013	NA	0.0043 ± 0.0014	NA
	Mokonzi		Nayembi	
	Study 1	Study 2	Study 1	Study 2
Nose wipe	0.008 ± 0.001	0.016 ± 0.002	0.008 ± 0.002	NA
Rough self-scratch	0.0008 ± 0.0004	0.001 ± 0.0004	0.0035 ± 0.0016	NA
Gentle self-scratch	0.0018 ± 0.00057	0.003 ± 0.0007	0.003 ± 0.00094	NA
	Vifijo		Zamba	
	Study 1	Study 2	Study 1	Study 2
Nose wipe	0.014 ± 0.002	NA	0.016 ± 0.002	0.029 ± 0.002
Rough self-scratch	0.0016 ± 0.0007	NA	0.0008 ± 0.0004	0.0018 ± 0.0006
Gentle self-scratch	0.0019 ± 0.00074	NA	0.00023 ± 0.00023	0.00039 ± 0.00026

.

Table S6: Post-hoc results of tested predictors in final models testing the effect of trial accuracy and hemispace. Simple contrasts were applied

**Study 1**						
Nose wipe ~ trial accuracy	Contrast	Estimate	SE	df	t-ratio	*P*
	Accuracy: Incorrect - correct	0.007	0.003	5846	2.447	0.014
Gentle self-scratch ~ trial accuracy + hemispace	Contrast	Estimate	SE	df	t-ratio	*P*
	Accuracy: Incorrect - correct	-0.014	0.026	220	-0.526	0.600
	Hemispace: left - right	-0.009	0.013	220	-0.747	0.456
Rough self-scratch ~ trial accuracy + hemispace	Contrast	Estimate	SE	df	t-ratio	*P*
	Accuracy: Incorrect - correct	-0.021	0.026	217	-0.903	0.367
	Hemispace: left - right	0.023	0.012	220	1.966	0.051
**Study 2**						
Nose wipe ~ trial accuracy	Contrast	Estimate	SE	df	t-ratio	*P*
	Accuracy: Incorrect - correct	0.011	0.002	4696	4.606	< 0.001
Gentle self-scratch ~ trial accuracy + hemispace	Contrast	Estimate	SE	df	t-ratio	*P*
	Accuracy: Incorrect - correct	-0.056	0.020	119	-2.886	0.005
	Hemispace: left - right	0.001	0.019	147	0.039	0.969
Rough self-scratch ~ trial accuracy * hemispace	Contrast	Estimate	SE	df	t-ratio	*P*
Hemispace: Left	Accuracy: Incorrect - correct	0.107	0.024	123	4.556	< 0.001
Hemispace: Right	Accuracy: Incorrect - correct	0.028	0.021	145	1.311	0.192
Accuracy: Incorrect	Hemispace: left - right	0.056	0.026	135	2.250	0.026
Accuracy: Correct	Hemispace: left - right	-0.022	0.018	141	-1.210	0.228

Table S7: Post-hoc results of tested predictors in final models testing the effect of trial accuracy and hemispace based on the subset of subjects that also participated in Study 2. Simple contrasts were applied.

**Study 1**						
Nose wipe ~ trial accuracy	Contrast	Estimate	SE	df	t-ratio	*P*
	Accuracy: Incorrect - correct	0.022	0.005	3342	4.009	< 0.001
Gentle self-scratch ~ trial accuracy + hemispace	Contrast	Estimate	SE	df	t-ratio	*P*
	Accuracy: Incorrect - correct	-0.016	0.038	100	-0.421	0.675
	Hemispace: left - right	-0.008	0.019	100	-0.438	0.662
Rough self-scratch ~ trial accuracy + hemispace	Contrast	Estimate	SE	df	t-ratio	*P*
	Accuracy: Incorrect - correct	-0.003	0.029	100	-0.107	0.915
	Hemispace: left - right	0.023	0.014	100	1.617	0.109
